# ENGAGING A COMMUNITY ADVISORY BOARD TO VALIDATE A CULTURALLY TAILORED INTERVENTION MODEL

**DOI:** 10.1093/geroni/igac059.1036

**Published:** 2022-12-20

**Authors:** Quinton Cotton, Gina Green-Harris, Juliet Chang, Laura Block, Andrea Gilmore-Bykovskyi

**Affiliations:** University of Wisconsin School of Medicine and Public Health, Madison, Wisconsin, United States; University of Wisconsin School of Medicine and Public Health, Madison, Wisconsin, United States; University of Wisconsin-Madison, Madison, Wisconsin, United States; University of Wisconsin-Madison School of Nursing, Madison, Wisconsin, United States; University of Wisconsin-Madison, Madison, Wisconsin, United States

## Abstract

National dementia research priorities call for interventional studies addressing the needs of vulnerable populations experiencing disparities and for greater inclusion of dementia care recipients and their family caregivers in research. While studies document disparities, high needs and experiences of African American dementia caregivers, there is limited translation of this knowledge into the development, implementation, and dissemination of dementia-specific culturally tailored interventions. Research methods that facilitate engagement capable of strengthening data collection and culturally congruent interpretation of caregiving perspectives are needed to inform intervention development. Drawing on a qualitative study utilizing Grounded Dimensional Analysis, we employed a longitudinal engagement method with a Community Advisory Board (CAB) to validate a culturally tailored crisis intervention model for African American dementia caregivers. We identified lessons learned through a review of transcripts, memos and research team discussions. CAB members identified language, family structure, cultural history, knowledge, and respect as important features for cultural tailoring.

